# Symptomatic COVID-19 in Pregnancy: Hospital Cohort Data between May 2020 and April 2021, Risk Factors and Medicolegal Implications

**DOI:** 10.3390/diagnostics13061009

**Published:** 2023-03-07

**Authors:** Marianna Maranto, Simona Zaami, Vincenzo Restivo, Donatella Termini, Antonella Gangemi, Mario Tumminello, Silvia Culmone, Valentina Billone, Gaspare Cucinella, Giuseppe Gullo

**Affiliations:** 1 Department of Obstetrics and Gynecology, Villa Sofia Cervello Hospital, University of Palermo, 90146 Palermo, Italy; 2 Department of Anatomical, Histological, Forensic and Orthopedic Sciences, “Sapienza” University of Rome, 00161 Rome, Italy; 3 Department of Health Promotion, Mother and Child Care, Internal Medicine and Medical Specialties, University of Palermo, 90127 Palermo, Italy; 4 Neonatal Intensive Care Unit, Villa Sofia Cervello Hospital, 90146 Palermo, Italy

**Keywords:** SARS-CoV-2 infection, pregnancy, vertical transmission, symptomatic COVID-19

## Abstract

Pregnancy does not appear to increase susceptibility to SARS-CoV-2 infection, but some physiological changes, such as the reduction of residual functional volumes, elevation of the diaphragm, and impaired cellular immunity, may increase the risk of severe disease and result in a higher risk of complications. The article’s primary objective is to evaluate the factors associated with symptomatic COVID-19 disease in pregnancy. The secondary objective is to describe maternal and neonatal outcomes and cases of vertical transmission of the infection. All pregnant women hospitalized with SARS-CoV2 infection were included in a prospective study in the UOC of Obstetrics and Gynecology, AOOR Villa Sofia—Cervello, Palermo, between May 2020 and April 2021. The patients who requested the termination of the pregnancy according to Law 194/78 were excluded. We included 165 pregnancies with a total number of 134 deliveries. Overall, 88.5% of the patients were asymptomatic at the time of admission and 11.5% were symptomatic. Of them, 1.8% of the patients required hospital admission in the intensive care unit. Symptoms occurrences were positively associated with the increase in maternal BMI (OR 1.17; *p* = 0.002), the prematurity (OR 4.71; *p* = 0.022), and at a lower birth weight (OR 0.99; *p* = 0.007). One infant tested positive for SARS-CoV2 nasopharyngeal swab; 11.4% of newborns had IgG anti SARS-CoV2 at birth; IgM was positive in 2.4% of newborns. There was no difference statistically significant difference in the vertical transmission of the infection among the group of symptomatic pregnant women and that of asymptomatic pregnant women.

## 1. Introduction

Coronavirus Disease-19 (COVID-19) is caused by the Severe Acute Respiratory Syndrome Coronavirus 2 (SARS-CoV-2) and includes several characterizations, from asymptomatic patients to respiratory failure, cardiac and cardiovascular complications, thromboembolic and inflammatory complications. Pregnancy does not appear to increase susceptibility to this infection, even if the entry into respiratory cells of SARS-CoV-2 is mediated by ACE2, and its expression increases during pregnancy, which may provide favorable conditions for SARS-CoV-2 infection [1].

Physiological changes during pregnancy, such as reduced functional residual volumes, diaphragm elevation, and altered cell immunity, may be at increased risk for severe disease, necessitating maternal intensive care unit admission, mechanical ventilation, and, in rare cases, extracorporeal membrane oxygenation [2,3]. Deaths have been reported equally in pregnant and non-pregnant women of reproductive age [4]. Among pregnant women, especially those who develop COVID-19 pneumonia, there is an increased risk of preeclampsia, preterm, and cesarean delivery due to fever and hypoxemia [5,6]. In this regard, it is worth mentioning a multinational cohort study which enrolled a total of 2130 women during the first phase of the pandemic, 706 of whom had COVID-19 and 1424 who were uninfected. Women diagnosed with COVID-19 were at higher risk of preeclampsia/eclampsia, serious infections, need for intensive care, maternal mortality, preterm delivery including iatrogenic, perinatal morbidity, and mortality. Although the presence of fever and dyspnea was associated with a higher risk of serious maternal and perinatal complications, asymptomatic patients were also at higher risk for maternal morbidity and preeclampsia [7,8,9,10].

Vertical transmission of SARS-CoV-2 appears to be limited, although it should not be ruled out altogether in light of the viral presence in placental villi and fetal membranes, which points to the possibility that the virus may be able to access the placenta and affect fetal development [1].

At any rate, the SARS-CoV-2-related cytokine storm could bring about higher morbidity and mortality rates in pregnant women, and possibly even pose a threat to the developing fetus and neonate, even in the absence of vertical viral transmission. For these reasons, more effective, evidence-based strategies, models, and targets need to be outlined, for the ultimate purpose of mitigating the impact of viral infection and improving maternal and fetal outcomes [1]. Furthermore, this evaluation should be useful in terms of raising awareness and clinical management abilities in the case of a new pandemic infection.

Information on SARS-CoV2-infected pregnancies is evolving rapidly and it is critical to collect data to plan for best practice. The main purpose of this study is to evaluate maternal and neonatal outcomes of pregnant women with SARS-CoV-2 infection. The secondary objective is to describe cases of vertical transmission of SARS-CoV2 infection.

## 2. Materials and Methods

### 2.1. Objectives

This study accounted for 165 pregnant women, with a total number of 134 deliveries, while 31 pregnant women were discharged before delivery due to improvement of clinical symptoms.

The patients were divided into groups based on the “NIH COVID-19 Treatment Guidelines” [11,12]:

✓ Asymptomatic or presymptomatic infection: positive test for SARS-CoV-2 but no symptoms;
✓ Mild illness: any signs and symptoms (e.g., fever, cough, sore throat, malaise, headache, muscle pain) without shortness of breath, dyspnea, or abnormal chest imaging;
✓ Moderate illness: evidence of lower respiratory disease by clinical assessment or imaging and a saturation of oxygen (SaO_2_) ≥ 94 percent at room temperature at sea level—severe illness: respiratory frequency > 30 breaths per minute, SaO_2_ < 94 percent on room air at sea level, ratio of arterial partial pressure of oxygen to fraction of inspired oxygen (PaO_2_/FiO_2_) < 300, or lung infiltrates > 50 percent;
✓ Critical illness: respiratory failure, septic shock, and/or multiple organ dysfunction.

### 2.2. Inclusion and Exclusion Criteria

In this retrospective study, all pregnant women hospitalized with SARS-CoV-2 infection in the Department of Obstetrics and Gynecology, Villa Sofia—Cervello Hospitals, Palermo, Sicily, between May 2020 and April 2021, were included. Pregnant women requiring termination of pregnancy as well as non-pregnant women hospitalized for other gynecological conditions were excluded.

### 2.3. Maternal and Fetal Outcomes

We collected personal and anamnestic data, reason and duration of hospitalization, duration of positivity to the nasopharyngeal molecular swab, symptoms, saturation, need for oxygen therapy or access to the intensive care unit, diagnostic tests and therapy. Fetal status was assessed by ultrasound and, in third-trimester pregnancies, with cardiotocography. We also evaluated pathologies of pregnancies and outcomes such as live birth, miscarriage, and stillbirth. For patients who gave birth with SARS-CoV-2 infection, data on delivery and any postpartum complications were collected.

### 2.4. Neonatal Outcomes

All newborns underwent a thorough review of their history, including gestational age, sex, birth weight, Apgar Score, type of feeding, length of hospitalization, symptoms for up to one week of age, such as respiratory distress, oxygen desaturation, feeding problems, fever, and/or seizures, heart rate anomalies thorough clinical examination, review of laboratory evaluations, rRT-PCR analysis of nasopharyngeal swab samples, and COVID-19 serology to determine SARS-CoV-2 status for those born to SARS-CoV-2-positive mothers.

### 2.5. Evaluation Vertical Transmission

All newborns from SARS-CoV-2 positive mothers were tested via a SARS-CoV-2 quantitative rRT-PCR nasopharyngeal swab at birth on day 3 and/or day 7 during their hospital stay. In case of positive result, neonates were re-tested on day 14.

### 2.6. Statistical Analysis

Socio-demographic and clinical characteristics of all the recruited pregnant women were summarized using frequencies and percentages. In order to evaluate the distribution of quantitative variables such as age, the skewness and kurtosis test was performed. Mean and standard deviation (SD) were chosen for the normal distribution of these variables, while median and interquartile range (IQR) were used for the non-normal distribution. The differences in quantitative variables normally and not normally distributed among pregnant with COVID-19 infection were evaluated, respectively, with the Student’s *t* test and with the Wilcoxon and Mann–Whitney test, while the Chi2 test was used for the qualitative variables.

Bivariable analyses were performed to assess the associations between factors allegedly linked to symptomatic (mild, moderate, and critical illness) COVID (Odds ratio (OR) with a confidence interval of 95%). The significant (*p* < 0.05) factors associated in bivariable analyses were run into a multivariable logistic regression model in order to identify predictors of symptomatic COVID-19. A *p*-value of <0.05 was considered statistically significant. Statistical analyses were performed using Stata/SE 14.2 (Copyright 1985–2015, StataCorp LLC, 4905 Lakeway Drive, College Station, TX 77845, U.S. Revision 29 January 2018).

## 3. Results

As shown in Table 1, 12 patients (7.3%) were hospitalized for COVID-related symptoms, 107 (64.9%) for obstetric reasons, 31 (18.8%) were new mothers transferred from other structures in the region due to a positive swab found in time of delivery, 9 (5.4%) were for abortion, and 6 (3.6%) were for other reasons.

At admission, 146 patients (88.5%) were asymptomatic, 8 (4.8%) had mild disease, 9 (5.5%) had moderate/severe disease, and 2 (1.2%) had critical disease.

During hospitalization, 11 (6.7%) patients needed oxygen therapy, while the remaining 154 (93.3%) did not. Finally, 3 patients (1.8%) required admission to the intensive care unit (Table 1).

The patients were then subdivided into two main groups: asymptomatic and symptomatic. As shown in Table 2, the symptomatic group showed a higher mean BMI than the asymptomatic group (33.9 vs. 28.9, *p* = 0.001); thus, the mode of delivery, preterm delivery and complications during hospitalization showed a statistically significant difference in the two groups (Table 2).

Regarding the 123 newborns admitted to the Department of Neonatology and Neonatal Intensive Care Unit, Villa Sofia Cervello Hospital, a statistically significant difference in birth weight was found between the group of symptomatic versus asymptomatic patients, as shown in Table 3.

Overall, 104 babies were born at term and 26 were born preterm; 66.6% of them were adequate for gestational age; 12.2% were small for gestational age (SGA) and 9.8% were large for gestational age (LGA). No neonatal respiratory distress was reported, and only 9% of newborns required noninvasive respiratory support at birth.

During their hospital stay, all infants remained asymptomatic, with normal temperature and vital parameters. Only one newborn was positive at nasopharyngeal swab for SARS-CoV2: the first SARS-CoV-2 test resulted positive at birth at their 25th hour of life. Repeated SARS-CoV-2 tests at 25, 48 h, 7 days, and 14 days of life were positive. The SARS-CoV-2 test resulted negative at 21 days of life. Soon after birth, the baby’s serology tested positive for both SARS-CoV-2 immunoglobulin (Ig)-G and Ig-M titers.

Overall, 11.4% (*n* = 14) of newborns had anti SARS-CoV2 IgG at birth; IgM was positive in 2.4% (*n* = 3) of newborns. There was no statistically significant difference in vertical transmission of the infection between the symptomatic and asymptomatic pregnant groups (Table 3).

A univariable analysis shows COVID-19 symptoms to be positively associated among maternal characteristics with increased maternal BMI (OR = 1.18, 95% CI 1.06–1.29, *p* value 0.002) and prematurity (OR 4.71; *p* = 0.022). After controlling for factors and statistical significance at multivariable analysis, only the unit increase of BMI (OR = 1.18, CI95% 1.04–1.35, *p* = 0.011) was associated with being symptomatic for COVID-19 among the maternal outcome (Table 4).

At univariable analysis, COVID-19 symptoms were positively associated among neonatal characteristics with birth weight (OR = 0.99, CI95% 0.99–1.00, *p* = 0.007). After controlling for other factors, COVID-19 symptoms of the mother were associated with the use of ventilation by the infants (OR = 26.95 CI95% = 1.26–574.29, *p* = 0.035) (Table 5).

## 4. Discussion

### 4.1. Risk-Factor Assessment

The article relies on data from the first cases of SARS-CoV2 positive pregnancies. The main results were that high BMI values were associated with a higher risk of symptomatic disease. These data are in agreement with the scientific literature that associates obesity with adverse outcomes from COVID-19 in the general population. Indeed, SARS-CoV-2 had the ability to gain entry into human cells by direct binding to ACE2 receptors on host cells [13]. Obese individuals have been found to be more susceptible to COVID-19 infection, which is likely due to the higher density of ACE2 in adipose tissue [14]. Furthermore, in animal models, tumor necrosis factor-α (TNF-α) is a multifunctional cytokine expressed in adipose tissue capable of influencing insulin-induced signaling and preventing glucose transporter type 4 (GLUT-4) expression, which gives rise to higher levels of free fatty acids (FFA), and worsening insulin resistance [15]. Immune inflammation pathways can be triggered by overly high levels of FFAs via several signaling pathways, ultimately leading to TNF-α, interleukin-6 (IL-6), leptin, and resistin [16], all of which play a direct role in the differentiation of monocytes into activated M1 macrophages. Inflammatory cytokines, active oxygen radicals, and nitric oxide (NO) can originate from M1 macrophages, and can negatively impact the endogenous immune response to pathogens [16]. The inflammatory response caused by obesity thus results in more pronounced cell aggregation and higher levels of cytokine production.

Nutritional status therefore has a major role in the development of COVID-19 complications, hospitalization length, and mortality rates, as pointed out by research findings from diverse populations [17]. Ultimately, obesity has been linked to higher rates of major complications due to its considerable impact on immune responses. In particular, an interesting review article has pointed to obesity as a considerable factor in the likelihood of incurring severe COVID-19 complications and the need for ICU admission, intubation, and even higher mortality rates. Such evidence makes it necessary to keep overweight and obese patients under close observation and monitoring at all times [18].

The second fundamental aspect which seems to emerge from the multivariate analysis is the linkage between symptomaticity and prematurity, and consequently low weight at birth.

Recent findings arising from an analysis of data gathered between April to May 2017 to 2019 and April to May 2020 pointed to a lower rate of prematurity (from 5.31% to 4.91%, *p* < 0.01). Furthermore, a decrease in the rate of prematurity was still observed after the end of lockdown (from June to September 2020) for singleton pregnancies. However, among the 1752 SARS-CoV-2-positive patients with singleton pregnancies, a higher prematurity rate was reported in 2020 than in 2017 to 2019 (9.93% vs. 5.32%; *p* < 0.01), regardless of the severity of prematurity. On the other hand, a lower prematurity rate was reported in uninfected or untested in 202 patients compared to those who gave birth in the 2017–2019 period (4.67% vs. 5.32%; *p* < 0.01), irrespective of prematurity severity [19].

A lower rate of preterm births during the COVID-19 pandemic has been reported by various sources [20,21,22,23], although it is worth noting that such an overall decrease does not account for SARS-CoV positive and negative patients, unlike the present study. In any case, some studies have reported that such a decrease was limited to deliveries to white patients residing in more affluent neighborhoods, and deliveries at non-outpatient care facilities; such findings may be due to the fact that COVID-19 response measures may have benefited women with more indicators of advantage [24]. A recent noteworthy retrospective cohort study has focused on the clinical manifestations, complications, and maternal–fetal outcomes in women with SARS-CoV-2 infection during delivery and divided patients into two groups: symptomatic and asymptomatic. Compared to asymptomatic patients, symptomatic pregnant women at the time of delivery were found to have slightly higher, though not significant, preterm delivery and cesarean section rates, in addition to lower neonatal birthweights and Apgar score, [25].

As for the high rate of caesarean sections, a retrospective review of case records in India compared outcomes (cesarean section rate, maternal and neonatal ICU admission, and feto-maternal mortality) in positive and negative pregnant women at delivery. Similar to our results, considerably higher cesarean section rates were reported among women with COVID-19. Furthermore, viral RNA was detected in the cord blood and nasopharyngeal swab of one infant [26].

Other studies also point to cesarean section as the most widespread delivery modality in parturient women with COVID-19. In particular, another Indian study accounting for 44 women undergoing cesarean section during the study period, with elective and emergency surgeries of 22 each, showed that no indication other than COVID-19 status was reported in 13 out of 44 patients [27].

In order to prevent the host COVID-19 complication herein laid out, the anti-SARS-CoV-2 vaccination is a safe and effective tool even in pregnancy. In this regard, an interesting mathematical model proposed by an Indian group that studied the transmission dynamics was associated with the decrease of COVID-19, underlining the importance of non-pharmaceutical interventions and vaccination as a strategy for the control of COVID-19 [28]. However, a part of the population of pregnant women still shows vaccine hesitancy, for which suitable counseling by gynecologists is certainly a valuable option worth pursuing [29].

A systematic review of a small sample of 6 early pandemic studies shows that, although vertical transmission of severe acute respiratory syndrome arising from coronavirus infection has so far been ruled out, and maternal and neonatal outcomes have been favorable overall, preterm delivery rates by cesarean section are still worrisome [30]. In any case, COVID-19, which is linked to respiratory insufficiency in late pregnancies, can undoubtedly give rise to a complex clinical scenario [6,31].

A review centered around 36 research studies has focused on deliveries in 203 SARS-CoV-2 positive pregnant women. Rather similar levels of disease severity in pregnant as opposed to non-pregnant women were reported. The majority of patients, 68.9%, ultimately gave birth via cesarean section, with COVID-19 status as the sole common indication [32].

As for the management of newborns at our hospital, the decision to separate newborns is necessarily made on a case-by-case basis, and is shared and agreed upon by mothers and the healthcare professionals based on a thorough risks vs. benefits evaluation. Mother and newborn were separated in the case of maternal severe clinical symptoms, or after surgery in cases of caesarean section, due to the impossibility of taking care of the newborn independently.

Mothers were counseled prior to discharge about home isolation and precautions, according to guidelines from the Italian Society of Neonatology. Clinical follow-up for infants was provided remotely and a repeat test for SARS-CoV-2 was administered 7 or 14 days after discharge, and then at 1 month after discharge.

Only one newborn tested positive at our facility. Furthermore, the mother wore a face mask throughout the hospital stay, had no skin-to-skin contact with the baby, no direct breastfeeding, no visitors (including parents) were allowed during the newborn’s first 14 days of life, and strict droplet isolation precautions took place. All such precautions notwithstanding, the newborn tested positive for SARS-CoV-2 at birth. In other studies, SARS-CoV-2 positive infants were also observed, but no definitive evidence of vertical transmission remains because available data are still insufficient [30]. Several studies now show that the SARS-CoV-2 genome can be detected in umbilical cord blood and placenta at term, and infants demonstrate elevated levels of SARS-CoV-2 specific IgG and IgM antibodies [33,34]. Although specimens of placental tissue or amniotic fluid found positive for any pathogen are considered a diagnostic sign of maternal infection, further confirmatory testing is needed before it can be deemed a sign of neonatal congenital infection [35]. Hence, it stands to reason that even though SARS-CoV-2 has been reportedly found by RT-PCR in the placenta, as reflected by various findings, a positive RT-PCR test in the fetus/neonate does not necessarily follow [36,37,38]. By the same token, positivity detected in the amniotic fluid does not necessarily entail fetal positivity. Even though RT-PCR in amniotic fluid have detected the presence of SARS-CoV-2 in a relatively limited number of case reports, not all infants were confirmed to be infected [39,40,41,42]. By virtue of such findings, a positive SARS-CoV-2 assay on amniotic fluid or placenta alone is not enough to provide a reliable level of confirmatory proof as to actual in-utero infection. As far as the contamination of umbilical cord blood is concerned, that is thought to possibly take place because of cross-contamination with maternal blood during sample collection, or blood cells from the mother getting into the fetal bloodstream through the placenta over the course of gestation, or most commonly, maternal blood cells getting into fetal circulation during labor, due to contractions of the uterus [43,44,45]. In light of such dynamics, confirmatory testing via fetal/neonatal peripheral blood sample or testing of another sterile or non-sterile sample is needed in addition to PCR.

It is likely that studies centered around placental tissue are the most significant in terms of providing insight as to SARS-CoV-2 vertical transmission. A Brazilian study on five pregnant women infected with SARS-CoV-2 before vaccination who gave birth to a stillborn child investigated placental alterations compared to a prepandemic sample. It is worth noting that RT-PCRq found SARS-CoV-2 RNA in three out of five placentas at least two to twenty weeks following primary pregnancy infection symptoms. Moreover, immunoperoxidase assays showed SARS-CoV-2 spike protein in all placental samples. Ultrastructural aspects of the infected placentas showed similar alteration patterns between the samples, with predominant characteristics of apoptosis and detection of virus-like particles [46].

### 4.2. Medicolegal Implications

In light of the risk factors associated with COVID-19 in pregnancy, both in terms of maternal and neonatal outcomes, it is worth briefly elaborating on the medicolegal repercussions that might arise from non-compliance with specific evidence based recommendations, guidelines, and best practices. The need to take into account the host of immunological changes which occur during pregnancy, over the third trimester especially, stems from the fact that such adjustments make women more vulnerable and more likely to develop major severe symptoms from SARS-CoV-2 infection; similar dynamics have also been shown with previous similar epidemics such as severe acute respiratory syndrome (SARS) and Middle East respiratory syndrome (MERS) [47,48]. COVID-19 vaccines based on mRNA have been shown to pose no safety concerns during pregnancy or breastfeeding, and they do not affect fertility [49]. A possible element complicating the decision-making process and the implementation of suitable treatment options is due the fact that COVID-19 during pregnancy often shows signs and symptoms similar to those in non-pregnant patients [50,51], although one systematic review found that pregnant and recently pregnant people were less likely to manifest fever, cough, dyspnea, and myalgia than non-pregnant females of reproductive age [52]. When assessing pregnant symptomatic people without fever, it is worth bearing in mind that it may be difficult to differentiate between several COVID-19 manifestations and common pregnancy symptoms, such as nausea, shortness of breath, and fatigue. Providing thorough counseling to pregnant patients as to the risks of COVID-19 infection is also key: the increased risk for severe disease from SARS-CoV-2 during pregnancy ought to be discussed and recommendations for the effective protection from infection should be given [53].

If a pregnant patient should need hospitalization due to COVID-19 infection, it ought to be at a hospital capable of guaranteeing maternal and fetal monitoring should the need arise. COVID-19 in pregnancy should be managed by ensuring fetal and uterine contraction monitoring, based on gestational age, whenever deemed advisable. In addition, delivery planning should be adequately designed and implemented on a case-by-case basis by relying on a multidisciplinary, team-based approach that may include consultation with obstetric, maternal-fetal medicine, infectious disease, pulmonary-critical care, and pediatric specialists, whenever deemed necessary. Provable and documented compliance with recommendations and evidence-based criteria outlined and released by scientific societies and institutions (such as the Centers for Disease Control and Prevention [54,55], the American College of Obstetricians and Gynecologists [56], and the Society for Maternal-Fetal Medicine [57], among others) can greatly contribute to ensuring that care for pregnant patients with COVID-19 is delivered in a viable fashion, from a medicolegal perspective, in order to shield healthcare professionals from negligence-based malpractice allegations in case of adverse outcomes [58,59,60]. Novel telemedicine-based methods of providing care and counseling to pregnant women need to take into account the relevant norms and regulations [61,62], as well as the unique complexities that such innovative practices entail from a legal and ethical standpoint [63,64].

## 5. Conclusions

The present study is based on the identification of patients at greater risk of contracting a symptomatic form of COVID 19 in order to reduce the onset of complications and, consequently, stem the increase in preterm deliveries, with possible sequelae in the short, medium and long term. Overall, findings from the present study support the claim that neonates born to mothers with confirmed or suspected SARS-CoV-2 are mostly asymptomatic, and therefore their status is not associated with worse clinical outcomes. However, neonatal critical illness is still a possibility; administering a nasopharyngeal swab at least at 24 h after birth and monitoring the infants for possible symptoms for 14 days after birth are necessary precautions and major contributors to medicolegal viability in case of adverse outcomes, as is long-term follow-up. Parents must be thoroughly counseled and directly involved in the decision-making process with options for rooming in, skin-to-skin contact, and breastfeeding with appropriate protective equipment, taking into account the organization of the hospital that hosts the pregnant patient and the newborn.

## Figures and Tables

**Table 1:** Descriptive analysis.

		Total *n* = 165
Reason for admission	COVID-19 symptoms	12 (7.3%)
	obstetrics	107 (64.9%)
	puerperium	31 (18.8%)
	miscarriage	9 (5.4%)
	other	6 (3.6%)
Symptomaticity	Asymptomatic or presymptomatic infection	146 (88.5%)
	Mild illness	8 (4.8%)
	moderate/severe illness	9 (5.5%)
	critical illness	2 (1.2%)
Oxygen therapy	yes	11 (6.7%)
	not	154 (93.3%)
Admission to intensive care	yes	3 (1.8%)
	not	162 (98.2%)

**Table 2:** Maternal descriptive analysis.

		Total *n* = 165	Symptomatic *n* = 19	Asymptomatic *n* = 146	*p*<
Age (median, IQR)		30 (19–44)	33 (29–35)	30 (26–35)	0.2
Nationality	Italy	141 (85.5%)	16 (11.3%)	125 (88.7%)	0.9
	Other	24 (14.5%)	3 (12.5%)	21 (87.5%)	
BMI (mean, SD)		29.4 (5.4)	33.9 (6.9)	28.9 (4.9)	0.001
Previous respiratory pathologies		4 (2.4%)	1 (25%)	3 (75%)	0.37
Previous autoimmunitary pathologies		13 (7.9%)	3 (23.1%)	10 (76.9%)	0.15
Previous hematological pathologies		10 (6.1%)	2 (20%)	8 (80%)	0.35
Smoke	yes	17 (10.3%)	1 (5.9%)	16 (94.1%)	0.815
	not	76 (46.1%)	10 (13.2%)	66 (86.8%)	
Pregnancy outcome	Caesarean section	65 (39.4%)	9 (13.8%)	56 (86.2%)	<0.001
	Spontaneous delivery	67 (40.6%)	0 (0%)	67 (100%)	
	Operative delivery	2 (1.2%)	0 (0%)	2 (100%)	
	Evolving pregnancy	15 (9.1%)	9 (60%)	6 (40%)	
	Miscarriage	10 (6.1%)	0 (0%)	10 (100%)	
	Ectopic pregnancy	2 (2.1%)	0 (0%)	2 (100%)	
Preterm birth	not	104 (63.0%)	5 (4.8%)	99 (95.2%)	0.014
	yes	26 (15.7%)	5 (19.2%)	21 (80.8%)	
Weeks to delivery, mean (SD)		36.5 (7.7)	37.1 (7.7)	36.4 (8.0)	0.8
Complications during hospitalization	none	142 (86.6)	11 (7.7%)	131 (92.3%)	<0.001
	Post-partum hemorrhage	5 (3%)	0 (0%)	5 (100%)	
	Pulmonary embolism	2 (1.2%)	1 (50%)	1 (50%)	
	Deep vein thrombosis	1 (0.6%)	0 (0%)	1 (100%)	
	Infections	2 (1.2%)	1 (50%)	1 (50%)	
	Hypertension in the puerperium	1 (0.6%)	0 (0%)	1 (100%)	
	Metrorrhagia in the puerperium	2 (1.2%)	0 (0%)	2 (100%)	
	Anemia	1 (0.6%)	0 (0%)	1 (100%)	
	other	8 (4.9%)	5 (62.5%)	3 (37.5%)	

Missing data: age = 3, BMI = 38, previous pathologies = 2, smoke = 72, pregnancy outcome = 3, preterm birth= 35, complications during hospitalization = 1.

**Table 3:** Neonatal descriptive analysis.

		Total *n* = 123	Symptomatic *n* = 11	Asymptomatic *n* = 112	*p*<
Birth weight (mean, SD)		3085 (584)	2610 (698)	3132 (553)	0.004
Weight according to gestational age	AGA	82 (66,6%)	6 (7.3%)	76 (92.7%)	0.74
	SGA	15 (12.2%)	2 (13.3%)	13 (86.7%)	
	LGA	12 (9.8%)	1 (8.3%)	11 (91.7%)	
Ventilation	not	96 (78%)	6 (6.25%)	90 (93.75%)	0.084
	yes	9 (7.3%)	2 (22.2%)	7 (77.8%)	
Nasopharyngeal swab positivity	not	117 (95.1%)	11 (9.4%)	106 (90.6%)	0.75
	yes	1 (0.8%)	0 (0%)	1 (100%)	
IgG positivity	not	50 (40.6%)	4 (8%)	46 (92%)	0.48
	yes	14 (11.4%)	2 (14.3%)	12 (85.7%)	
IgM positivity	not	61 (49.6%)	6 (9.8%)	55 (90.2%)	0.57
	yes	3 (2.4%)	0 (0%)	3 (100%)	
Intensive care	not	107 (87%)	10 (9.3%)	97(90.7%)	0.95
	yes	11 (8.9%)	1 (9.1%)	10 (90.9%)	

Missing data: weight according to gestational age = 14, nasopharyngeal swab positivity = 5, IgG positivity = 59, IgM positivity = 59, intensive care = 5.

**Table 4:** Maternal univariable and multivariable analysis of factors associated to symptomatic COVID-19.

		Crude OR	IC95%		*p*	Adjusted OR	IC95%		*p*
Median age > 30 vs. <30 years		2.46	0.83	7.26	0.103	0.42	0.06	3.17	0.402
Nationality	Italy	ref							
	Other	1.12	0.30	4.16	0.870				
BMI per unit increase		1.17	1.06	1.29	0.002	1.18	1.04	1.35	0.011
Previous respiratory pathologies yes vs. no		2.78	0.27	28.29	0.387				
Previous autoimmunitary pathologies yes vs. no		2.70	0.67	10.91	0.163				
Previous hematological pathologies yes vs. no		2.14	0.42	10.97	0.361	2.39	0.14	41.72	0.55
Smoke yes vs. no		0.64	0.22	1.86	0.415				
Preterm birth		4.71	1.25	17.75	0.022	5.66	0.77	41.24	0.087

**Table 5:** Neonatal univariable and multivariable analysis of factors associated with symptomatic COVID-19.

		Crude OR	IC95%		*p*	Adjusted OR	IC95%		*p*
Birth weight (mean)		0.99	0.99	1.00	0.007	0.99	0.99	1.00	0.074
Weight according to gestational age	AGA	ref				ref			
	SGA	1.95	0.35	10.72	0.443	1.04	0.06	18.80	0.979
	LGA	1.15	0.13	10.49	0.900	3.27	0.14	77.66	0.464
Ventilation		4.33	0.73	25.56	0.105	26.95	1.26	574.29	0.035
IgG positivity		1.92	0.31	11.74	0.482				
Intensive care		0.80	0.19	3.41	0.769	0.01	0.01	2.94	0.110

## Data Availability

The datasets used and/or analyzed during the current study are available from the corresponding author on reasonable request.
